# Mechanistic Insights into Dinitrogen Reduction to Ammonia in Light-Controlled Nanocrystal:Nitrogenase Complexes

**DOI:** 10.1021/acs.accounts.5c00763

**Published:** 2026-04-03

**Authors:** Ritika Sharma, Florence Mus, Lauren M. Pellows, Peter J. Dahl, David W. Mulder, Zhi-Yong Yang, Paul W. King, Gordana Dukovic, Lance C. Seefeldt, John W. Peters

**Affiliations:** † Department of Chemistry and Biochemistry, 6187 University of Oklahoma, Norman, Oklahoma 73019, United States; ‡ Department of Chemistry and Biochemistry, 4606 Utah State University, Logan, Utah 84322, United States; § Biosciences Center, 53405 National Laboratory of the Rockies, Golden, Colorado 80401, United States; ∥ Department of Chemistry, 1877 University of Colorado Boulder, Boulder, Colorado 80309, United States; ⊥ Renewable and Sustainable Energy Institute (RASEI), 1877 University of Colorado Boulder, Boulder, Colorado 80303, United States; # Materials Science and Engineering, 1877 University of Colorado Boulder, Boulder, Colorado 80303, United States

## Abstract

Developing systems that can efficiently capture photon energy and convert this energy into fuels and chemicals requires understanding how to assemble molecular components with diverse functions into complete systems possessing selectivity and efficiency in directing charge carriers to catalytic reactions. There are many challenges to achieving this goal. One promising approach is the development of hybrid systems that combine semiconductor nanocrystals (NCs) for light capture and enzymes as efficient catalysts.

Such biohybrid systems capitalize on the tunable electronic and optical properties of NCs while leveraging the unmatched specificity and efficiency of enzymes in catalyzing chemical reactions, thereby offering opportunities to surpass the limitations of each component alone. Here, we focus on recent progress in developing a biohybrid system that combines CdS NCs for photon capture with the enzyme nitrogenase to accomplish light-driven dinitrogen (N_2_) reduction to ammonia (NH_3_). Integrating light-harvesting materials with biological catalysts requires a deep understanding of NC properties, protein stability, and electron transfer (ET), making it an inherently multidisciplinary problem.

The reduction of N_2_ to NH_3_ is a challenging reaction, with a high demand in both agriculture and industrial chemical production. This reaction is intrinsically energy intensive, due to the need to activate the N≡N triple bond. The current standard industrial approach to N_2_ reduction, the Haber–Bosch reaction, obtains the necessary energy input from fossil fuels, whereas biological systems capable of N_2_ reduction utilize the hydrolysis of ATP as their energy source. Replacing these costly, energy-intensive inputs with renewable light energy represents a critical step toward sustainable NH_3_ production.

Recent progress has demonstrated that semiconductor CdS NCs can be coupled to the catalytic component of nitrogenase, the MoFe protein, to form a biohybrid CdS NC:MoFe protein complex, enabling light-driven N_2_ reduction rather than energy input from fossil fuels or ATP. This illustrates how inorganic NCs can functionally replace the natural Fe protein partner, yielding a biohybrid catalyst that enables controlled electron delivery and provides not only light-driven NH_3_ production but also new approaches for probing enzyme catalytic function.

The CdS NC:MoFe protein biohybrid system enables light-initiated electron delivery at ambient temperature, as well as temperatures below freezing, allowing for stabilization and spectroscopic characterization of key reaction intermediates. These findings highlight how photochemical biohybrids can serve as both functional catalysts and mechanistic probes. Beyond studies of the nitrogenase mechanism, studies of the CdS NC:MoFe system reveal how variables such as NC size, electrostatic binding interactions, and sacrificial electron donors (SEDs) govern complex stability, charge transfer efficiency, and catalytic performance.

In addition, studies of nitrogenase and the high activation barrier for N_2_ reduction are enabling investigations of new and interesting questions regarding the properties and limitations of NC biocatalysis. In this Account, we describe the key features of CdS NC:MoFe protein biohybrids and the parameters for optimal light-driven N_2_ reduction, and how controlling ET with light illuminates the path to new insights into the nitrogenase mechanism.

## Graphical abstract

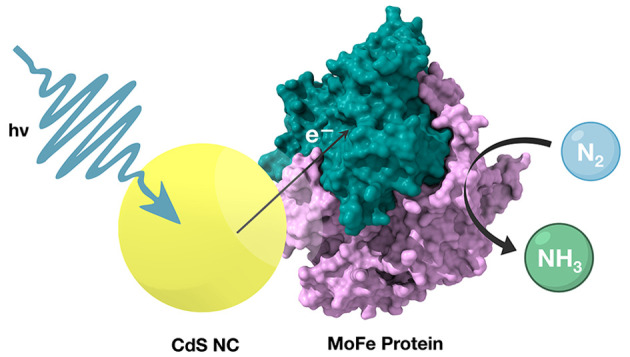

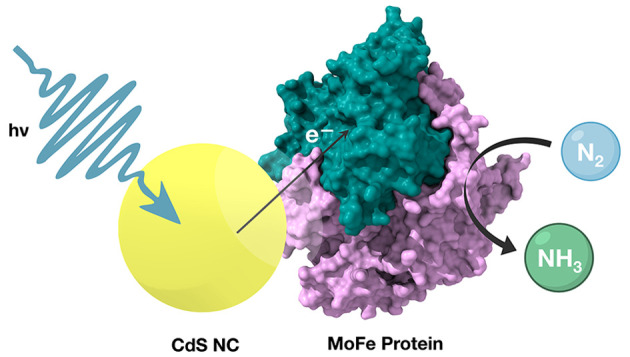

## Key References

-

Brown, K. A. ; Harris, D. F. ; Wilker, M. B. ; Rasmussen, A. ; Khadka, N. ; Hamby, H. ; Keable, S. ; Dukovic, G. ; Peters, J. W. ; Seefeldt, L. C. ; King, P. W. Light-driven dinitrogen reduction catalyzed by a CdS:nitrogenase MoFe protein biohybrid. Science 2016, 352 (6284), 448–450.27102481 10.1126/science.aaf2091 ^1^ CdS nanorods were shown to photosensitize nitrogenase MoFe protein, replacing ATP-dependent Fe protein electron transfer; light harvesting drove ammonia formation with turnover rates near those of the natural enzyme. This proof-of-concept biohybrid underpins subsequent photochemical N_2_-reduction work.
-

Chica, B. ; Ruzicka, J. ; Kallas, H. ; Mulder, D. W. ; Brown, K. A. ; Peters, J. W. ; Seefeldt, L. C. ; Dukovic, G. ; King, P. W. Defining intermediates of nitrogenase MoFe protein during N_2_ reduction under photochemical electron delivery from CdS quantum dots. J. Am. Chem. Soc. 2020, 142 (33), 14324–14330.32787260 10.1021/jacs.0c06343 ^2^ Under low photon-flux photodriven conditions, CdS quantum dots were used to populate and identify a two-electron (E_2_) intermediate of nitrogenase. The study clarified the initial electron-transfer steps and demonstrated that electron flux controls the photochemical reaction cycle.
-

Ruzicka, J. L. ; Pellows, L. M. ; Kallas, H. ; Shulenberger, K. E. ; Zadvornyy, O. A. ; Chica, B. ; Brown, K. A. ; Peters, J. W. ; King, P. W. ; Seefeldt, L. C. ; Dukovic, G. The kinetics of electron transfer from CdS nanorods to the MoFe protein of nitrogenase. J. Phys. Chem. C 2022, 126 (19), 8425–8435.^3^ Transient absorption spectroscopy quantified electron transfer kinetics from CdS nanorods to the MoFe protein, revealing quantum efficiencies consistent with quantum yields of NH_3_ and H_2_ formation. Electron transfer depended strongly on nanorods size, with diameters >4.2 nm showing no detectable transfer.
-

Vansuch, G. E. ; Mulder, D. W. ; Chica, B. ; Ruzicka, J. L. ; Yang, Z.-Y. ; Pellows, L. M. ; Willis, M. A. ; Brown, K. A. ; Seefeldt, L. C. ; Peters, J. W. ; Dukovic, G. ; King, P. W. Cryo-annealing of photoreduced CdS quantum dot:nitrogenase MoFe protein complexes reveals the kinetic stability of the E_4_(2N2H) intermediate. J. Am. Chem. Soc. 2023, 145 (39), 21165–21169.37729189 10.1021/jacs.3c06832PMC10557137 ^4^ Photoreduced CdS:MoFe protein biohybrids were cryo-annealed to trap the four-electron E_4_(2N2H) state. EPR analysis showed this intermediate is kinetically stable and that H_2_ oxidative addition proceeds via an associative mechanism. This work provides mechanistic insight into the E_4_ Janus intermediate.
-

Pellows, L. M. ; Willis, M. A. ; Ruzicka, J. L. ; Jagilinki, B. P. ; Mulder, D. W. ; Yang, Z.-Y. ; Seefeldt, L. C. ; King, P. W. ; Dukovic, G. ; Peters, J. W. High affinity electrostatic interactions support the formation of CdS quantum dot: nitrogenase MoFe protein complexes. Nano Lett. 2023, 23 (22), 10466–10472.37930772 10.1021/acs.nanolett.3c03205 ^5^ Using microscale thermophoresis, this study demonstrated that CdS quantum dots bind to MoFe protein primarily through electrostatic interactions, with stronger affinities for smaller dots. Quantifying these interactions guides optimization of biohybrid assembly and electron transfer for efficient N_2_ reduction.
-

Dahl, P. J. ; Pellows, L. M. ; Yang, Z.-Y. ; Seefeldt, L. C. ; Peters, J. W. ; Dukovic, G. ; Mulder, D. W. ; King, P. W. Pre-steady-state kinetics of nanocrystal:molybdenum nitrogenase biohybrids reveals hole-scavenging efficiency is critical to N_2_ reduction. Cell Rep. Phys. Sci. 2025, 6 (8), 102732.^6^ A pre-steady-state kinetic model based on time-resolved EPR spectra of illuminated CdS:MoFe biohybrids revealed a new inactivation pathway, along with insight into the rate-limiting role of hole scavenging in the catalytic reaction for light-driven N_2_ reduction.

## 1 Introduction

Growing global energy needs demand technologies for efficient light capture and conversion into chemicals to support life and serve as energy carriers. This need has motivated the development of alternative strategies for *de novo* fuel production compatible with existing infrastructure. Regardless of the starting materials, the production of fuels requires low-potential electrons to reduce oxidized chemicals, forming H–H, C–C, C–H, and N–H bonds—the primary classes of bond-forming reactions in energy conversion. Nature offers a rich set of paradigms for such multielectron, low-potential redox chemistry through redox enzymes such as [FeFe]- and [NiFe]-hydrogenases;^7^ formate dehydrogenases (FDH);^8^ 2-oxoglutarate:ferredoxin oxidoreductase (OGOR);^9,10^ and nitrogenase,^1^ which mediate H–H, C–H, C–C, and N–H bond formation, respectively. These biocatalysts demonstrate that highly challenging reductive, bond-forming reactions can be accomplished in ambient conditions with remarkable specificity and efficiency.

An attractive option to meet the global energy challenge is to directly couple the capture of light energy, and excited state electron transfer, with the reduction of abundant oxidized chemicals, creating an array of reduced compounds that can serve multiple needs, including acting as energy carriers. The efficacy of such an approach relies on the coupling of an appropriate photosensitizer with an effective catalyst that produces fuels. In this context, semiconductor NCs—nanoscale semiconductor materials whose optical and electronic properties are governed by quantum confinement—including quantum dots (QDs) and nanorods (NRs), have emerged as versatile photosensitizers, providing a modular, tunable platform for driving redox chemistry under visible-light illumination.^11–13^ Upon photoexcitation, NCs generate long-lived electron–hole pairs with tunable redox potentials that can be matched to a wide range of catalytic reactions. When paired with sacrificial electron donors (SEDs) that efficiently quench photogenerated holes, these systems can sustain reductive chemistry by channeling excited-state electrons to molecular catalysts or enzymes. This general photochemical strategy has been widely applied to light-driven H_2_ evolution, CO_2_ reduction, and C–C bond formation, establishing NCs as a platform for coupling photoexcited carriers with multielectron catalysis.^13–16^

Coupling NCs with enzymes that catalyze oxidation–reduction reactions, such as hydrogenases,^7,70^ FDH,^17^ OGOR,^9,10^ and nitrogenases,^1^ is an approach to forming fuels with H–H, C–C, C–H, or N–H bonds. Enzymes have evolved to mediate multielectron redox transformations with exquisite specificity and efficiency. Recent work has demonstrated the successful integration of these biocatalysts with photosensitizers (Figure 1), yielding biohybrid systems capable of light-driven H_2_ evolution, CO_2_ reduction, and N_2_ fixation. For example, NC:hydrogenase assemblies achieve efficient photochemical H_2_ generation,^18^ whereas FDH or OGOR systems yield formate or 2-oxoglutarate generated from CO_2_ under illumination, respectively.^10,17^ Such biohybrid assemblies provide a conceptual framework for evaluating how light-driven charge generation can be matched to the kinetic and thermodynamic requirements of multielectron catalysis. In addition, light control of fuel-forming catalysts provides the means to synchronize electron transfer (ET) events and populate intermediates for interrogation, offering opportunities to probe catalytic mechanisms under non-steady-state conditions.

**Figure 1:**
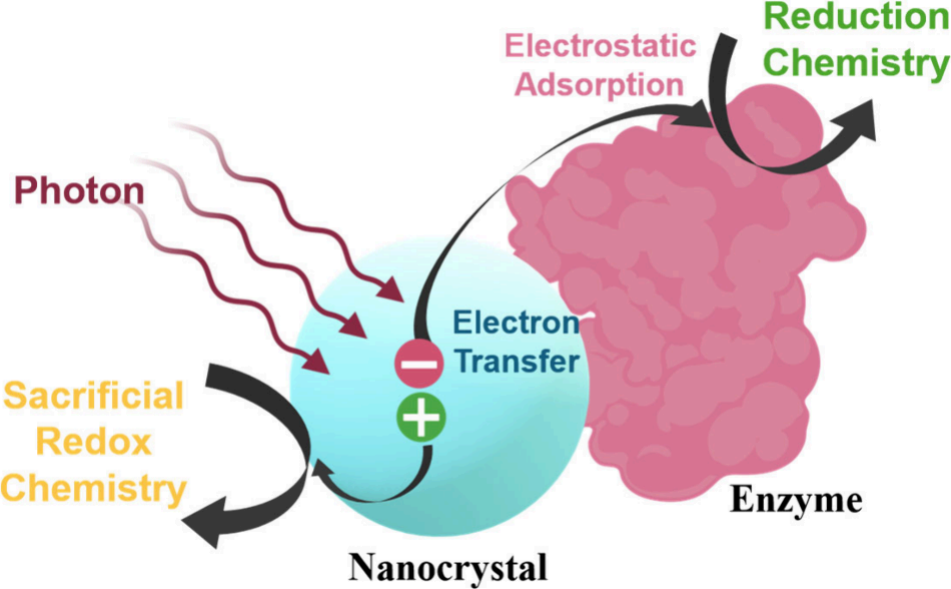
Schematic overview of light-driven nanocrystal (NC):enzyme biohybrids, enabling new photochemical routes for substrate reduction and fuel-forming reactions.

Among these transformations, biological dinitrogen (N_2_) reduction represents one of the most energetically demanding and mechanistically complex reactions. Nitrogenase is a complex metalloenzyme that naturally reduces N_2_ to ammonia (NH_3_) using adenosine 5′-triphosphate (ATP) hydrolysis as the energy source.^19–71^ Nitrogenase is comprised of two proteins: the Fe protein, the native electron donor, and the catalytically active MoFe protein.^19–71^ Replacing ATP-dependent Fe protein electron delivery with photochemical electron injection establishes a compelling strategy for light-driven studies of nitrogenase and motivates its use as a model system for exploring how NC photosensitizers interface with complex enzymes that catalyze difficult, multielectron reactions.^1^ In addition, coupling redox enzymes to light-harvesting materials provides a platform for dissecting fundamental aspects of enzyme catalysis, such as ET dynamics and catalytically active intermediate stabilization under photochemical control.

### 1.1 Early Studies and Proof of Concept

Early efforts to bypass the need for Fe-protein and ATP for nitrogenase substrate reduction used strong soluble reductants such as europium­(II) complexed with organic ligands,^20–24^ or covalently attached ruthenium bipyridine photosensitizers,^25,26^ all of which were demonstrated to deliver electrons to MoFe protein and its variants. These approaches provided valuable mechanistic insights but did not support N_2_ reduction or significant enrichment of reaction intermediates, thereby limiting their utility for probing the catalytic pathways of NH_3_ production.

A breakthrough was achieved when our team demonstrated light-driven N_2_ reduction using CdS NR:MoFe protein biohybrids under strict anaerobic conditions (Figure 2).^1^ In these experiments, 3.8 nm × 16.8 nm CdS NRs capped with 3-mercaptopropionic acid (MPA) were illuminated at 405 nm in the presence of millimolar concentrations of a SED, enabling photochemical electron delivery to the MoFe protein. Under these optimized conditions, NH_3_ was produced at a maximum turnover frequency (TOF) of ∼ 75 mol NH_3_ (mol MoFe protein)^−1^ min^–1^, corresponding to ∼ 63% of the maximum rate observed for the native Fe protein and ATP-driven system.^1^ Turnover numbers (TONs) exceeding 1.1 × 10^4^ mol NH_3_ (mol MoFe protein)^−1^ were achieved over 5 h, establishing that multiple catalytic cycles could be sustained photochemically.^1^

**Figure 2:**
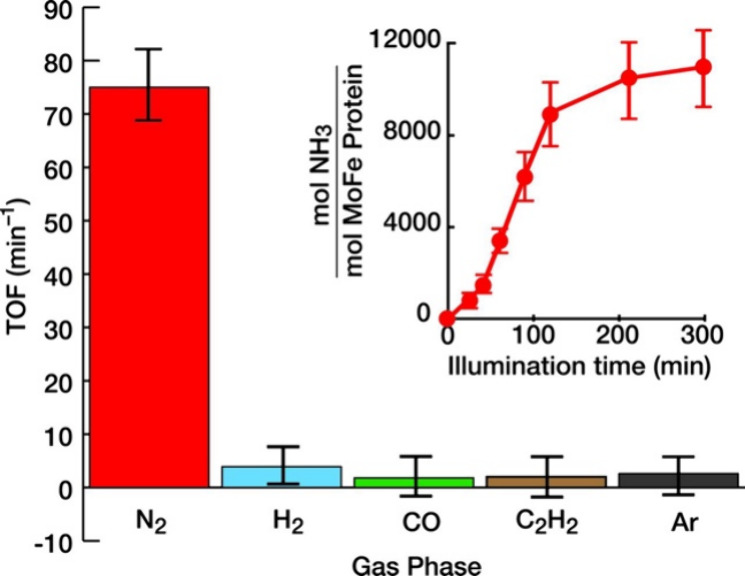
Turnover frequency (TOF) for NH_3_ formation under 100% N_2_ (red), with inhibition by H_2_ (cyan), CO (green), and C_2_H_2_ (brown). Ar atmosphere (gray) is a negative control. The inset depicts the time-dependent NH_3_ production under 100% N_2_. Adapted from ref 1. Copyright © 2016, The American Association for the Advancement of Science.

The quantum yield (QY) for NH_3_ formation under these conditions was estimated to be 3.3%, indicating that only a modest fraction of absorbed photons led to productive nitrogenase turnover even though the TOF approached the maximum reported for MoFe nitrogenase.^1^ This low QY value was hypothesized to reflect competition between productive electron injection into the enzyme and non-productive pathways, including electron–hole recombination, and H_2_ production by the enzyme. As such, QY as a measure of photochemical efficiency is complementary to TON as a measure of catalytic robustness.

Importantly, these early experiments also delineated boundaries on effective system operations. In this original work, efficient NH_3_ formation was sensitive to a variety of complex variables, including illumination power, NC-to-enzyme ratios, and hole-scavenging efficiency, with deviations resulting in diminished activity, increased H_2_ evolution, or loss of colloidal stability. Together, TON and QY provide complementary performance descriptors, highlighting both the promise and intrinsic efficiency limitations of the photochemical system. Collectively, these findings demonstrated that photochemical electron delivery can functionally replace ATP-dependent Fe protein-driven ET, while also revealing intrinsic limitations imposed by electron-loss pathways.

The broader significance of this proof-of-concept is that it establishes a light-driven, externally controllable entry point for delivering electrons to the MoFe protein, in contrast to the tightly coupled, continuous ATP-dependent Fe protein cycle. In practice, illumination intensity and duration define the rate and extent of electron injection, enabling access to non-steady-state regimes that are difficult to achieve in the ATP-dependent Fe protein system. Subsequent studies exploiting this light-controlled electron delivery have demonstrated that photon flux can modulate electron accumulation on the MoFe protein, enabling pre-steady-state trapping of discrete catalytic intermediates and revealing competition between productive ET and non-productive electron-loss pathways.^2,4–6^ These experiments provide a direct experimental basis for using photochemical systems to interrogate mechanistic features of nitrogenase catalysis.

In parallel, quantitative analyses of TONs, QYs, and charge-separation dynamics have clarified how efficiency and apparent durability of the catalytic system are limited by NC:enzyme coupling, hole-scavenging efficiency, and recombination processes, rather than by intrinsic enzyme instability.^1,3,5,6,27^ These insights, developed in later sections, demonstrate how photochemical biohybrids can serve as mechanistic tools and reveal performance bottlenecks relevant to scalable photocatalytic platforms.

## 2 Semiconductor Nanocrystals as Photosensitizers

Semiconductor NCs, typically 2–10 nm in diameter, exhibit quantum confinement effects allowing size-, shape-, and composition-dependent absorption, emission, and tuning of conduction and valence bands. The energies of the conduction band (CB) and valence band (VB) dictate the redox potentials. Upon photoexcitation, an electron is excited from the VB to the CB, leaving behind a hole in the VB. For quantum-confined NCs, the exact energy levels of the VB and CB is determined by the NC size due to confinement effects. Specifically, for 3.8 nm diameter CdS NRs (band gap = 2.72 eV), the CB (estimated at −0.8 V vs NHE) is sufficiently negative to reduce iron–sulfur clusters, while the VB (estimated at +1.9 V vs NHE) is oxidizing enough for hole scavenging from an array of compounds, such as hydroquinone (HQ) or ascorbic acid (AA).^1^

NCs are effective photosensitizers due to their high absorption cross sections, relatively long-lived excited states, and tunable surface chemistry.^14,28^ Surface ligands regulate solubility and electrostatic interactions between the NC and enzyme, promoting binding.^14,28^ In CdS NC:nitrogenase biohybrids, short, negatively charged carboxylate ligands such as MPA were found to promote productive ET by simultaneously maintaining colloidal stability and enabling electrostatic association with positively charged regions on the MoFe protein surface.^1,3,5^ Once bound, an excited electron can undergo several relaxation pathways following photoexcitation. It can recombine with the hole, be trapped at the surface, or be transferred to the enzyme.^11,29^ Transient absorption spectroscopy (TAS) directly probes the charge dynamics of NC excited states. The addition of the enzyme opens a new relaxation pathway for the excited electron, resulting in a shortening of the NC excited-state lifetime (Figure 3). Through analytical modeling, the rate constants for the different relaxation pathways can be determined from fitting the models to TAS data.^11,14,29^ In CdS NR:MoFe protein assemblies, the rate constants for interfacial ET (*k*_ET_), recombination (*k*_rec_), and surface trapping (*k*_tr_) were found to be on comparable time scales between 1 and 100 ns, resulting in competition between these processes that is quantified by the quantum efficiency of electron transfer (QE_ET_).^3^ QE_ET_ is the probability of photoexcited ET to the enzyme.

**Figure 3:**
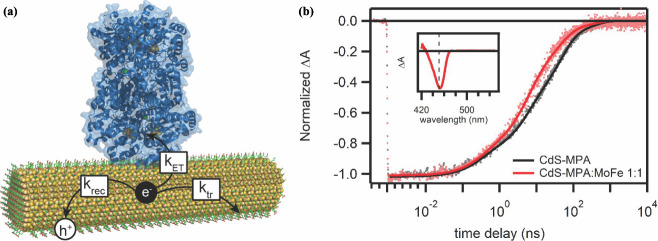
Transient absorption spectroscopy and electron decay pathways in CdS:MoFe biohybrids. (a) Schematic of photoexcited electron-decay pathways in CdS nanorod (NR):MoFe protein (PDB: 1M1N) complexes (*k*_rec_, rate constant for recombination; *k*_ET_, rate constant for interfacial electron transfer; *k*_tr_, rate constant for surface trapping). (b) Transient absorption bleach at 435 nm for 3.8 nm × 17.6 nm CdS NRs capped with MPA with and without MoFe protein (1:1 ratio). Solid lines represent fits to the kinetic model. Inset: ΔA spectrum after 1 ns after 400 nm excitation. Adapted from ref 3. Copyright © 2022, American Chemical Society.

In photochemical systems such as the NC:nitrogenase biohybrids, where the rate of photoexcitation is much lower than the rate constants for intrinsic relaxation processes within the NC, the rate of electron injection into the enzyme is the product of the excitation rate and QE_ET_.

$$rate\;of\;electron\;injection\propto QE_{ET}\times excitation\;rate$$ 1

Based on eq 1, the QE_ET_ also defines the upper limit on the QY of product formation, i.e. the QY that would be observed if other processes were not rate-limiting. If QE_ET_ equals 100%, in principle, all photoexcitation leads to an ET event, and the electron injection rate is limited only by the excitation rate. As the QE_ET_ decreases, only a fraction of the excitations result in an ET event, decreasing the rate of electron injection. Thus, the upper limit on QY can be improved by increasing the QE_ET_, which can be achieved through different strategies such as increasing the rate of interfacial ET or suppressing trapping and recombination. For example, one can envision that by changing NC properties, such as using shorter surface ligands, the electronic coupling can be enhanced, increasing the QE_ET_.^30,31^ Other processes, such as hole scavenging by an SED and back ET from the enzyme to a hole in the NC, can also become rate-limiting and directly affect the QY.^30^ Donors such as AA and HQ, which quench photogenerated holes, help decrease the probability of electron–hole recombination by removing the hole before the next excitation event, are essential for sustained activity. Together, these observations suggest that improving QY requires coordinated optimization of multiple NC parameters and hole-scavenging efficiency, rather than enhancing any single parameter in isolation.

## 3 Assembly of Nanocrystal:Enzyme Biohybrids

### 3.1 Binding Interactions and Thermodynamics

The surface chemistry of NCs plays a pivotal role in this assembly. CdS NCs are typically capped with mercaptocarboxylic acid ligands, such as MPA, which, at neutral pH, confers a net negative surface charge via terminal carboxylate groups. These surface charges serve two distinct but complementary functions: (1) electrostatic repulsion between adjacent NCs, which suppresses NC aggregation and precipitation and (2) binding to MoFe protein through electrostatic attraction to positively charged regions on the MoFe protein surface.

Direct measurement of NC:nitrogenase interactions is challenging because it requires strict anaerobic conditions to maintain nitrogenase stability. Because of this, we established a microscale thermophoresis (MST) workflow tailored for such complexes, employing sealed anaerobic capillaries to preserve the stability of the fluorescently labeled MoFe protein and its fluorescence.^32–34^ This innovation enabled, for the first time, quantitative determination of dissociation constants (K_d_) for MoFe protein:CdS QD interactions, representing a critical step in dissecting biohybrid assembly.

Using anaerobic MST, evidence for electrostatically driven binding was obtained from an ionic-strength dependence study (Figure 4d).^5^ At low ionic strength, the longer Debye screening length preserved repulsive QD:QD interactions, maintaining colloidal dispersion, while also sustaining attractive MoFe protein:CdS QD interactions. At high ionic strength, the electrostatic screening shortens the Debye length, simultaneously weakening MoFe protein:CdS QD attraction and reducing interparticle repulsion between QDs. This dual effect promoted QD aggregation and diminished binding to the MoFe protein, leading to a weaker apparent affinity.

**Figure 4:**
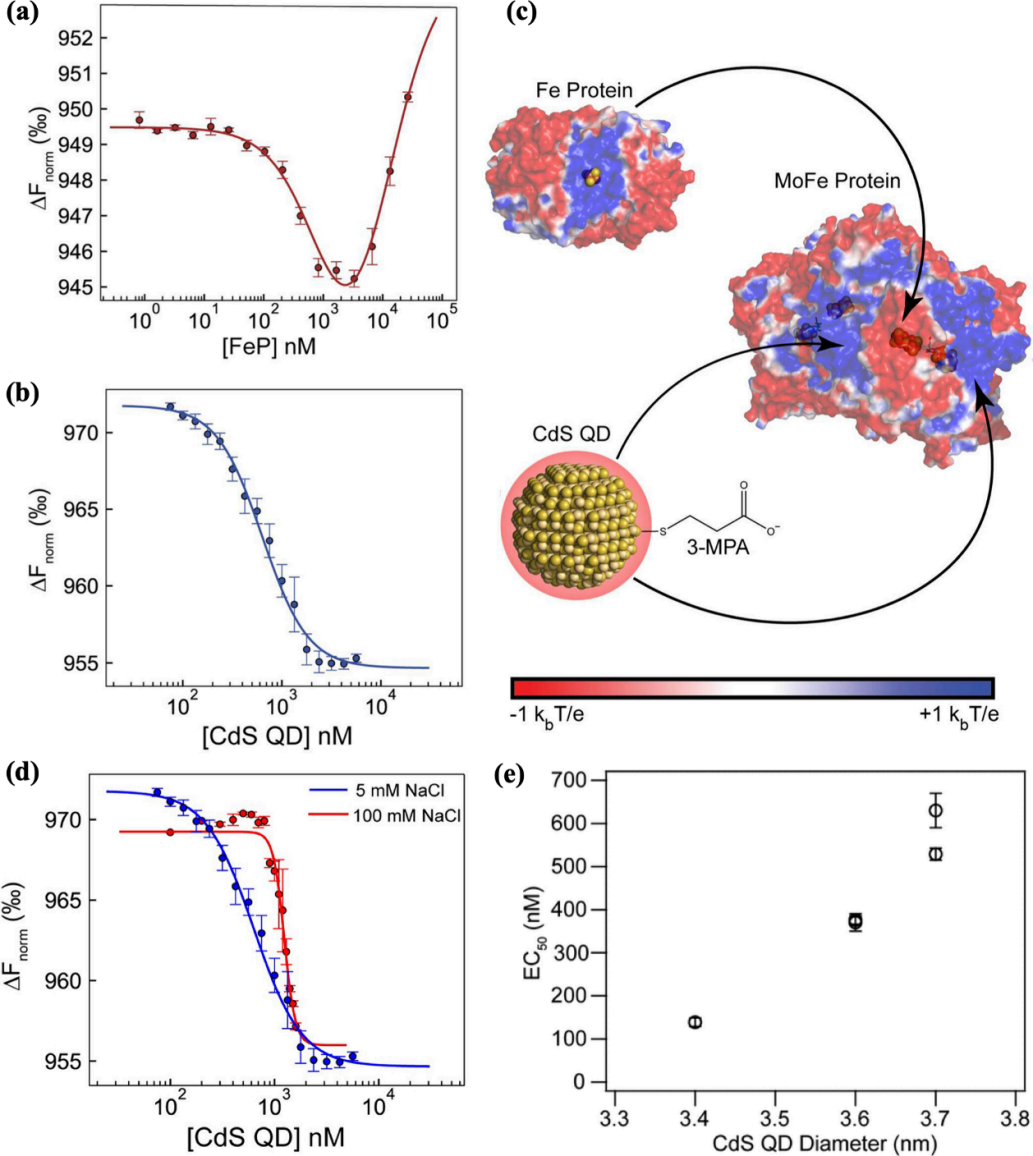
Microscale thermophoresis (MST) binding curves and electrostatic governing QD:MoFe complexation. (a) MoFe protein:Fe protein binding (K_d_ ≈ 920 ± 130 nM). (b) MoFe protein:CdS QD binding (K_d_ ≈ 640 ± 40 nM). (c) Electrostatic surfaces of MoFe (PDB: 1M1N), Fe protein (PDB: 1FP6), and CdS QD (3-MPA capped), showing proposed binding sites. (d) Binding curves at 5 mM versus 100 mM NaCl, indicating weaker binding with higher ionic strength. (e) K_d_ increases with QD size, showing reduced affinity at larger diameters. Adapted from ref 5. Copyright © 2023, American Chemical Society.

We then compared the binding strength of CdS QDs capped with MPA to the native electron donor and found that CdS QDs bind to the MoFe protein with affinities (K_d_ ≈ 640 ± 40 nM) comparable to the native Fe protein (K_d_ ≈ 920 ± 130 nM) (Figures 4a and 4b).^5^ Interestingly, these interactions exhibited markedly different binding behavior, as reflected in the shape of the binding curves. The Fe protein binding curve was fit to a two-site binding model and showed negative cooperativity, consistent with its well-established, regulated docking cycle in which binding of one Fe protein molecule induces conformational changes that disfavor a subsequent binding events.^35,36^ In contrast, the CdS QD binding curve was fit to a multi-site model and exhibited positive cooperativity, suggesting that initial QD association promoted additional binding interactions, likely through multivalent electrostatic interactions with the MoFe protein.

The MoFe protein contains two metal clusters—the P-cluster, the native electron injection site, and FeMo-co, the active site for substrate binding and reduction. The electrostatic potential surface map of the MoFe protein reveal discrete positively charged surface patches proximal to both the P-cluster and the FeMo-co (Figure 4c). These patches span only a few nanometers in lateral extent, as inferred from crystallographic structures and electrostatic calculations, placing a geometric constraint on productive NC association. Consistent with this interpretation, an NC size series revealed strong binding for QDs with diameters ≤ 3.7 nm, whereas particles ≥ 4.0 nm exhibited no detectable binding (Figure 4e).^5^ This threshold likely arises from a geometric mismatch between the larger QDs and the surface area of these positively charged surface patches, which may limit multivalent electrostatic contact and stable binding.

### 3.2 Electron Pathway and Structural Organization

Nitrogenase catalysis proceeds through sequential electron–proton transfers (ET/PT) that advance the FeMo-co active site through reaction intermediates that are represented as E_n_ states in the Lowe–Thorneley (LT) kinetic scheme (Figure 5). In the native system, the P-cluster, an [8Fe-7S] metallocluster, serves as an intermediate electron relay between the Fe protein and the FeMo-co in native catalysis. The P-cluster operates according to a proposed deficit-spending model, in which it transiently donates an electron to the FeMo-co first before being reduced by the Fe protein bound to the MoFe protein (Figure 6).^37–40^ As electrons and protons accumulate on FeMo-co, the cofactor reaches the four-electron reduced state, E_4_(4H), commonly referred to as the Janus intermediate.^41^ At this stage, reductive elimination (re) of H_2_ is coupled to N_2_ binding, yielding the E_4_(2N2H) intermediate, which then proceeds through subsequent proton-coupled electron transfer (PCET) steps consistent with either distal or alternating reduction pathway proposed for nitrogenase catalysis.^42–50^ Oxidative addition (oa) of H_2_ can revert E_4_(2N2H) back to E_4_(4H) (Figure 5).^2,4,46,42–48^ In ATP-driven turnover, several key intermediates have been trapped, and their interconversions have been studied in detail.^46,51^ However, several intermediates still cannot be trapped with the traditional freeze-trapping method. In contrast, photochemical electron delivery enables access to pre-steady-state regimes and intermediate trapping at cryogenic temperatures, providing a powerful alternative route for mechanistic interrogation.

**Figure 5:**
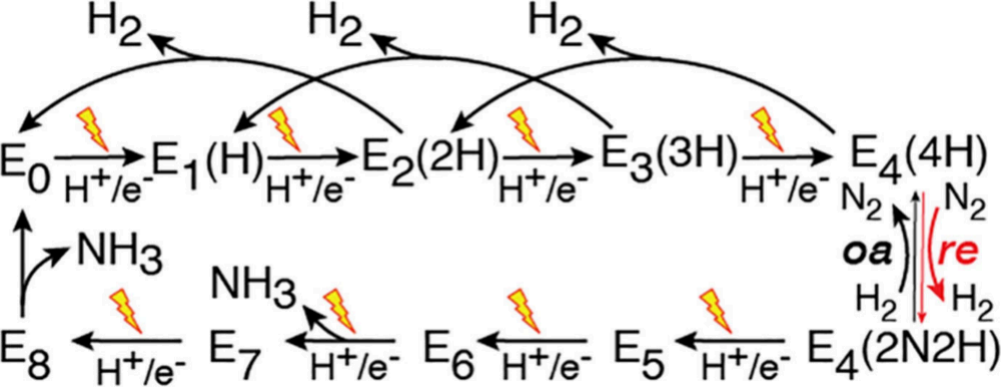
Lowe–Thorneley kinetic mechanism of the ET/PT steps that lead to the sequential accumulation of one-electron reduced intermediate states, or “E-states”, of the MoFe protein identified as E_0_ to E_8_. Even E-states in MoFe protein are paramagnetic and can be detected by electron paramagnetic resonance (EPR) spectroscopy. Photochemical electron delivery in NC:MoFe protein complexes (represented by lightning bolts) provides a unique strategy for probing the catalytic intermediates and mechanistic steps of the cycle. Adapted from ref 4. Copyright © 2023, The Authors. Published by American Chemical Society. Available under a CC-BY 4.0.

**Figure 6:**
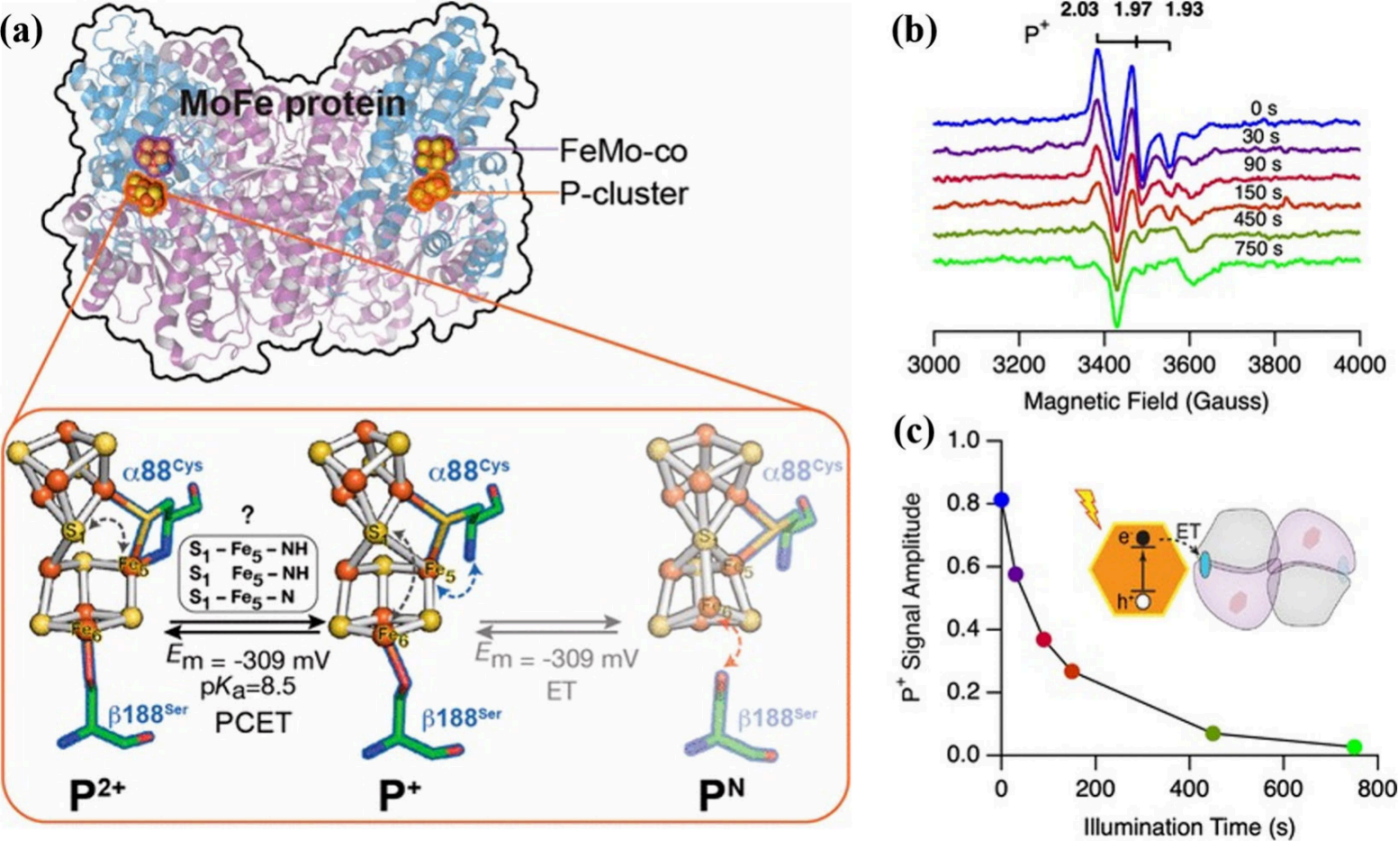
(a) Redox-dependent ligand coordination changes of the MoFe protein P-cluster during P^2+^→P^+^→P^N^ transitions. Adapted from ref 53. Copyright © 2022, The Authors. Published by American Chemical Society. Available under CC-BY 4.0. (b, c) EPR spectra and time-dependent P-cluster P^+^ signal evolution of CdS:β-188Cys MoFe complexes under illumination at 274 K. Adapted from ref 2. Copyright © 2020, The Authors. Published by American Chemical Society.

By developing a method for controlled, light-driven electron paramagnetic resonance (EPR) spectroscopy, Chica et al. were able to follow the reduction of the E_0_ state and the accumulation of the two-electron E_2_ state using low-photon-flux conditions.^2^ By increasing the photon flux, Pellows et al. drove accumulation of more reduced FeMo-co states (E_4_ and beyond).^2,4,6,52^ These light-driven EPR results demonstrate that photoexcited electrons can reduce the FeMo-co of the MoFe protein. To explore whether the P-cluster participates in photochemical electron delivery from CdS NCs to FeMo-co, experiments employed the β-188Cys MoFe variant, in which a cysteine (Cys) substitution alters the P-cluster electronic environment, stabilizing the P^+^ oxidation state and enhancing its spectroscopic signature.^2^ Under low photon-flux conditions, 405 nm illumination of CdS:β-188Cys MoFe complexes resulted in attenuation of the P^+^ EPR signal, consistent with reduction of the P-cluster to P^N^ rather than oxidation to P^2+^ (Figures 6b and 6c). These data demonstrate that photogenerated electrons can reduce oxidized P-cluster and FeMo-co states.^2^ These results indicate that both P-cluster and FeMo-co are accessible sites for photochemical electron injection, although the electron flow between them remains unknown.

The geometry of the NC:MoFe protein complexes remains challenging to define due to protein flexibility and possible heterogeneous binding modes. Nevertheless, electrostatic surface potential maps of the MoFe protein identify positively charged regions proximal to both the P-cluster and FeMo-co, suggesting that NC binding could occur near either site depending on orientation and local interaction.^3^ Thermodynamic considerations are consistent with this possibility—the CdS NRs CB (∼ −0.85 V vs NHE) provides sufficient driving force to either the P-cluster (ΔV > −0.5 V) or FeMo-co (ΔV > −0.4 V).^3,53^ However, it is important to emphasize that these arguments establish thermodynamic feasibility rather than experimentally demonstrated ET pathways.

Taken together, current data support a working model in which photochemical electron delivery may access electron entry points distinct from those enforced by Fe protein docking, but do not yet establish whether native conformational gating is fully bypassed. This distinction raises a key open question for future studies: the implications of NC-mediated electron delivery decouples ET from Fe protein-dependent conformational control.

### 3.3 Hole Scavenging and Charge Separation

System performance depends on the balance between NCs photophysics and catalytic demand. In an analogous system of NCs with hydrogenase, it was shown that, under certain conditions, hole transfer can limit photochemical product formation efficiency.^30^ To understand the effects of hole scavenging in the CdS NC:MoFe protein system, Clinger et al. performed assays with 3.4 nm MPA-capped CdS QDs and MoFe protein in MOPS buffer (pH 7.0) under 405 nm illumination, quantifying product yields across different SEDs. It was found that the effects of hole scavenging on CdS NC:MoFe protein biohybrid catalysis are highly dependent on the SED identity. Within this framework, SED identity and concentration strongly influenced photocatalytic rates of substrate reduction (Figures 7a–7d). The SED sodium dithionite (DT) supported maximal rates near ∼ 50 mM. Still, it showed diminished activity at higher concentrations, likely due to changes of the ionic strength of the solution and its effect on the formation of CdS QD:MoFe protein biohybrid complex rather than to its intrinsic electron-donating ability. In contrast, AA and triethanolamine (TEOA) maintained activity up to 100 mM, whereas HQ peaks near 50 mM.^27^ These trends mirror observations in nanoparticle-based photoredox systems involving small-molecule catalysts,^14,28,31,52,54^ indicating that SEDs play a dominant role in governing hole-scavenging kinetics and colloidal stability rather than enzyme-specific chemistry.

**Figure 7:**
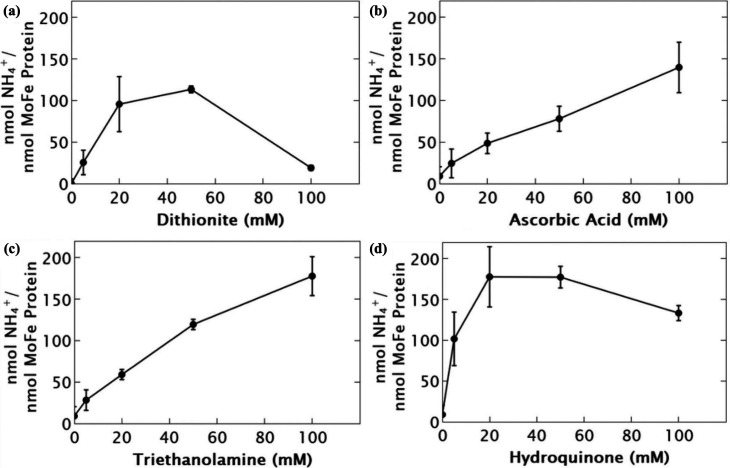
Product yields of CdS QD:MoFe protein across varying concentrations of sacrificial electron donors (SEDs). Reactions were carried out in 50 mM MOPS (pH 7.0) with 5 mM DT (except when DT alone was varied), under 405 nm illumination (30 mW, 1 h). Panels: (a) DT, (b) AA, (c) TEOA, and (d) HQ. Adapted from ref 27. Copyright © 2024, The Authors. Published by Elsevier Inc. Available under CC-BY 4.0.

## 4 Mechanistic Insights from Photochemical N_2_ Reduction

### 4.1 Pre-Steady-State Kinetics

Recent pre-steady-state kinetic studies by Dahl et al., which used time-resolved detection of intermediates by light-driven EPR, demonstrated that hole-scavenging efficiency directly influences electron accumulation on FeMo-co in CdS NC:MoFe biohybrids.^6^ Inefficient scavenging led to charge recombination and partial inactivation of the MoFe protein, whereas effective scavenger concentrations sustained electron flux and catalytic activity.^6^ Pre-steady-state kinetic modeling incorporated photochemical oxidation and inactivation pathways into the LT framework (Figure 8), explicitly testing how electron injection, hole scavenging, and recombination shape the E_n_ distribution.^6^ Here, the model serves as a hypothesis-testing framework, allowing experimental trends to rule out scenarios inconsistent with observed intermediate populations. For example, inefficient hole scavenging predicts an increased rate of oxidation (E_n_→E_n‑1_) and generation of off-pathway inactive states (E_0_→X), consistent with experimental observations.^6^ These studies reveal that light-controlled systems can exhibit a broader, more detailed distribution of reaction intermediates than are typically observed with steady-state, biochemically driven enzymatic turnover, which reflects a complex interplay between photochemical and catalytic processes.

**Figure 8:**
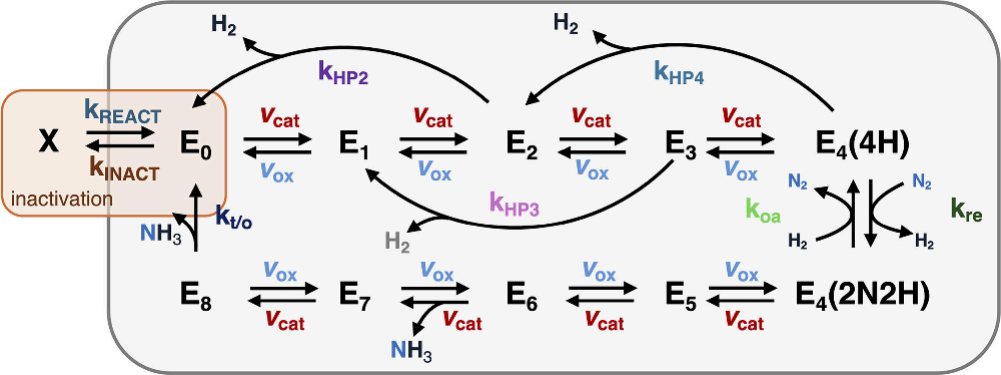
Schematic of a modified Lowe–Thorneley kinetic scheme adapted for photochemical N_2_ reduction. The model includes an off-path inactivation pathway (E_0_→X) alongside sequential electron–proton transfer steps (v_cat_, v_ox_), hydride protonation (*k*_HP_), reductive elimination (*k*_re_), oxidative addition (*k*_oa_), and E_8_→E_0_ turnover (*k*_t/o_). Inactivation and reactivation are governed by rate constants *k*_INACT_ and *k*_REACT_, respectively. Adapted from ref 6. Copyright © 2025, The Authors. Published by Elsevier Inc. Available under CC-BY 4.0.

Together, these results highlight that photochemical electron delivery enables controlled access to specific regions of the catalytic landscape. Photochemical approaches afford an experimental handle for populating, stabilizing, and interrogating reaction states that are otherwise transient or kinetically masked in the native ATP-driven system leading to new mechanistic insights.

### 4.2 Electron Flux and Intermediate Trapping

Light-controlled electron delivery alters the timing and regulation of electron input relative to Fe-protein cycling, facilitating experimental access to the accumulation of catalytic intermediates. As discussed above, reduced FeMo-co states can be populated via photoreduction and pre-steady-state trapping at cryogenic temperatures.^2,4,6,52^ Cryo-annealing experiments demonstrated that trapping the E_4_(2N2H) state slows catalytic turnover by minimizing H_2_ formation, thereby stabilizing the N_2_-bound intermediate for an extended duration (decay rate ≈ 10^6^ min).^4^ Importantly, this effect is not simply due to generalized kinetic slowing at low temperature. Rather, cryogenic photoreduction suppresses H_2_ evolution and reveals reversible interconversion between E_4_(4H) and E_4_(2N2H). These observations are consistent with the long-proposed “Janus model”, in which reductive elimination of H_2_ is coupled to N_2_ binding at the E_4_ junction.

Low-temperature photoreduction experiments followed by cryo-annealing also helped resolve the dynamic spin-state landscape of the P-cluster (Figures 9a and 9b). At 231 K, photoreduction of the MoFe protein poised in the P^2+^ state led to attenuation of the P^2+^ signal and growth of axial (S = 1/2) P^+^ and high-spin (S = 7/2) P^+^ signals, along with suppression of a rhombic P^+^ signal that was observed at a higher illumination temperature. During subsequent dark annealing at 236 K, which allows relaxation toward thermodynamic equilibrium, the axial and high-spin signals decayed while the rhombic P^+^ species accumulated, indicating interconversion among P^+^ conformers. These observations are consistent with a three-step kinetic model in which spectroscopically distinct P^+^ species (P^+^_6.54_ and P^+^_1.89_, defined by their EPR g-values) serve as intermediates en route to the more stable P^+^_1.81_ state.^53^ Activation barriers inferred from temperature dependence and pH-dependent population shifts indicate that proton-linked structural rearrangement accompanies these electronic transitions, with lower pH favoring accumulation of specific P^+^ conformers. Thus, spin-state changes reflect distinct structural isomers of the P-cluster that may modulate ET kinetics through conformational gating (Figures 6b and 6c).^37,56^ The precise mechanistic role of protonation in these transitions remains unresolved.^53^

**Figure 9:**
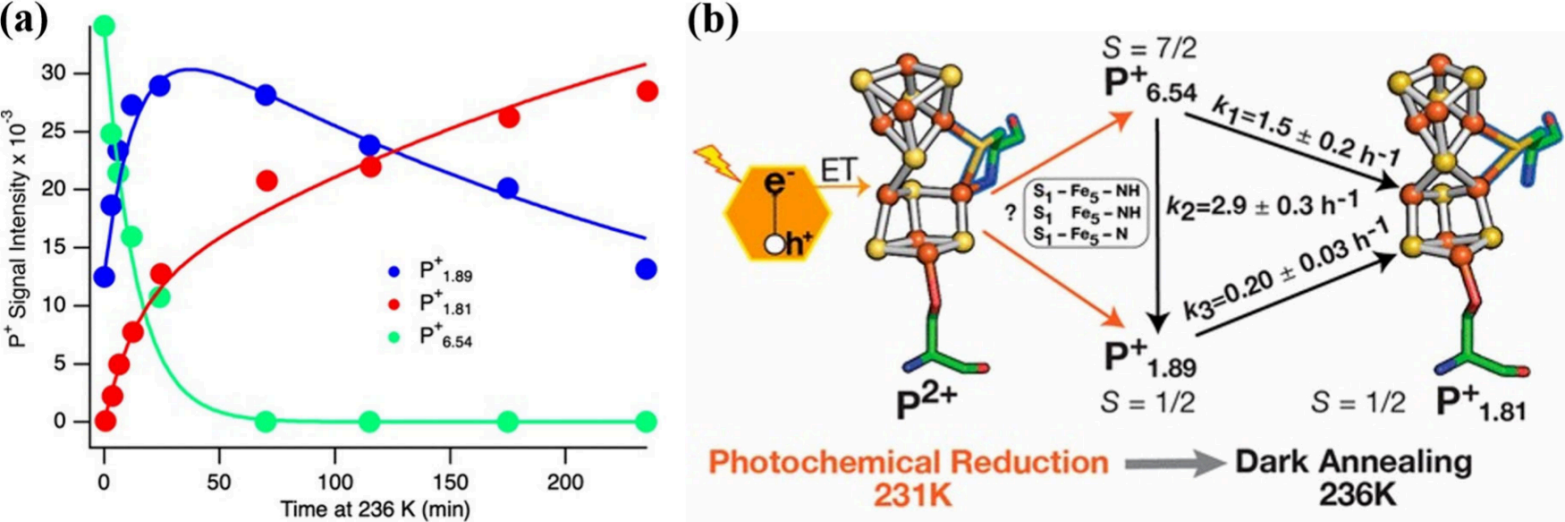
Spectroscopic characterization of MoFe protein P-cluster states upon illumination and dark annealing of CdS:MoFe protein complexes. (a) Kinetic fits of changes in P^+^ EPR signal intensities during dark annealing of CdS:MoFe protein complexes at 236 K performed after illumination at 231 K. Adapted from ref 53. Copyright © 2022, The Authors. Published by American Chemical Society. Available under CC-BY 4.0. (b) Schematic of P^2+^ photoreduction to distinct P^+^ conformers and their interconversion. Adapted from ref 53. Copyright © 2022, The Authors. Published by American Chemical Society. Available under CC-BY 4.0.

## 5 [FeFe]-Hydrogenase: Complementary and Parallel Insights

Studies on NC:[FeFe]-hydrogenase biohybrids reveal complementary mechanistic insights that parallel key questions in nitrogenase biohybrids, particularly regarding where electrons enter the enzyme, how electron relays shape downstream chemistry, and how photochemical electron delivery couples to PT. Ratzloff et al. showed that in CdSe QD:[FeFe]-hydrogenase I (CaI) complexes, photogenerated electrons enter via distal [4Fe-4S] clusters (F-clusters), which functions as an electron relay to the catalytic H-cluster.^18^ This provides a clear, experimentally validated example of NC-to-relay electron injection—an issue that remains under active investigation in nitrogenase systems (P-cluster vs FeMo-co entry).

EPR and rapid-scan FTIR spectroscopy confirmed that reduction of the oxidized H-cluster (H_ox_) to the one-electron reduced state (H_red_H^+^) proceeds through a PCET mechanism, with an activation enthalpy (ΔH^‡^) of ∼ 19 kJ mol^–1^ and a solvent kinetic isotope effect (KIE) of ∼ 2.5.^18^ While analogous PCET steps are inherently central to nitrogenase catalysis (multiple coupled electron/hole additions across E states), direct experimental isolation of individual PCET steps in nitrogenase remains challenging due to its multielectron complexity and conformational gating. Thus, hydrogenase biohybrids provide a tractable benchmark for establishing how photochemical electron delivery interfaces with coupled proton/electron chemistry.

Importantly, electron injection into F-clusters was also observed in the same enzyme lacking the H-cluster, consistent with a binding model specific to F-cluster electron injection.^18,52^ This “relay-first” injection behavior offers a concrete comparison point for nitrogenase: it motivates experimentally testable hypotheses whether photochemical injection similarly targets a relay (P-cluster) or can directly access the catalytic center (FeMo-co), depending on the NC binding geometry.

Together, these results establish [FeFe]-hydrogenases as tractable models for dissecting (i) how NC binding defines the electron-entry site, and (ii) how that entry site is kinetically coupled to proton transfer chemistry at the active site. In this sense, the hydrogenase system provides “parallel insights” by resolving, in a simpler enzyme, mechanistic features that are more difficult to isolate directly in nitrogenase.

Beyond a mechanistic comparison, hydrogenases can play a synergistic role in integrated biohybrid schemes. Whereas the MoFe protein specializes in multielectron N_2_ reduction it also generates H_2_ as a byproduct, hydrogenases catalyze rapid, reversible H_2_ oxidation to supply protons and low-potential electrons for reductive chemistry. This functional complementarity suggests opportunities for coupled systems in which H_2_ management and electron supply are designed to enhance overall photochemical efficiency.

## 6 Conclusions and Outlook

The integration of semiconductor NCs and metalloenzymes represents a powerful frontier in photocatalysis, merging the tunability of inorganic materials with the sophistication of biological catalysts. For nitrogenase, CdS NC biohybrids establish a non-ATP, light-driven mode of operation in which photochemical electron delivery functionally replaces Fe protein-mediated ET, enabling externally controlled electron flux and non-steady-state regimes. A key outcome of these studies is that photochemical nitrogenase populates canonical LT intermediates, including E_2_, E_4_, and the N_2_-bound E_4_(2N2H) state, and directly validates reversible H_2_ re/oa at the Janus junction. These shared intermediates demonstrate substantial conservation of the core catalytic chemistry at the FeMo-co. At the same time, photochemical operation bypasses the Fe protein-ATP cycle, altering how electrons are delivered and removing native conformational gating. Thus, while downstream chemistry appears conserved, the ordering and regulation of individual ET steps that lead to the formation of E-state intermediates may differ. As a result, this system provides a unique opportunity to interrogate whether and how ET gating contributes to the N_2_ reduction mechanism.

From a mechanistic point of view, light-driven operations clarify key constraints, including competition between interfacial ET, recombination, and surface trapping, and highlight the central roles of hole scavenging and photon flux in determining quantum efficiency and intermediate stability. Photochemical control reshapes experimental access to individual catalytic steps and loss pathways that are difficult to resolve under steady-state enzymatic turnover. From these systems, generalized principles emerge—electrostatic control of NC:enzyme binding, coordinated optimization of ET and hole scavenging, and judicious choice of sacrificial donors—that parallel those established in NC biocatalysis. Enzyme biohybrids are distinctive in that photophysical parameters can be directly correlated with well-defined catalytic intermediates.

Looking forward, challenges remain in achieving long-term colloidal stability, high interfacial ET efficiency, and robust hole-scavenging chemistries. Future efforts should prioritize non-toxic, earth-abundant semiconductors with broader visible absorption to expand spectral utilization. Furthermore, translating these successes into scalable systems can also benefit from innovations in material design and the development of model biohybrid structures to guide the engineering of molecular assemblies. Extending photochemical electron delivery strategies to nitrogenase isozymes (V- and Fe-only nitrogenases) and probing mechanistic intermediates under controlled photon flux will further clarify electron–proton coupling in N_2_ reduction. More broadly, NC:enzyme biohybrids advance sustainable routes to fuels and provide platforms for probing, controlling, and ultimately engineering complex biological catalysis using light.
